# Efficacy of bone defect therapy involving various surface treatments of titanium alloy implants: an in vivo and in vitro study

**DOI:** 10.1038/s41598-023-47495-w

**Published:** 2023-11-17

**Authors:** Boyang Wang, Yu Guo, Jiuhui Xu, Fanwei Zeng, Tingting Ren, Wei Guo

**Affiliations:** 1 Musculoskeletal Tumor Center, Peking University People’s Hospital, No. 11 Xizhimen South Street, Beijing, 100044 People’s Republic of China; 2 Beijing Key Laboratory of Musculoskeletal Tumor, Beijing, People’s Republic of China

**Keywords:** Cell biology, Cell adhesion, Materials science

## Abstract

Multiple surface treatment methods for titanium alloy prostheses, widely used in orthopedics, are available; however, these can affect bone integration and regeneration efficiency. In this study, through cell and animal experiments, we devised seven bone implant categories of Ti6Al4V based on surface preparation and post-processing technology (polishing, grit-blasting, fine titanium spraying, coarse titanium spraying, electron beam melting [EBM] printing, selective laser melting [SLM] printing, and post-processed SLM printing) and imaged each microscopic surface structure with a scanning electron microscope (SEM). Mechanical testing revealed excessive post-processing damaged the mechanical properties of the implants. In vitro, human bone marrow mesenchymal stem cells (hBMSCs) were cultured with implants, and the morphology of the cells adhering to the implant surface was observed using SEM and confocal laser scanning microscopy. Cell Counting Kit-8 (CCK-8) semi-quantitatively determined cell activity, indirectly reflecting the proliferation of hBMSCs. Alizarin red and alkaline phosphatase experiments assessed osteogenic differentiation. In vivo, experiments utilized the New Zealand rabbit femoral condyle bone defect model to assess bone regeneration and integration using micro-computed tomography, Van Giesen staining, and Masson staining. We found that 3D-printed implants with regular pore structures were more conducive to hBMSC osteogenic differentiation, while the presence of metal powder on NPT-SLM-printed implants hindered such differentiation. The post-treatment SLM scaffold surface may have some residual semi-melted powder; however, these powder residues have no significant effect on cell activity and differentiation. Surface treatment (grit-blasting and titanium spraying) of planar structures can enhance hBMSC adhesion but does not necessarily promote their differentiation. The framework structure of 3D printing may affect the osteogenic differentiation of hBMSCs, and for SLM-printed implants, excessive pursuit of a “powderless” state will damage the mechanical properties of the implant.

## Introduction

Titanium (Ti) and its alloys, known for their exceptional corrosion resistance and mechanical strength, have become widely used materials for fabricating clinical implants^1,2^. However, as medical technology advances, the expectations for implants have evolved, demanding more than durability and reliability. For example, the osteogenic activity of bone integration and efficiency in bone repair implants are now subject to higher standards.

Ti alloys, being inert metals, do not inherently possess bioactivity. However, researchers have discovered that by modifying the surface of an alloy, the desired functionalities can be induced via coatings. For example, magnesium (Mg), zinc (Zn), and tantalum (Ta) can impart osteogenic activity to implants^3,4^, while silver (Ag) and copper (Cu) have antibacterial properties^5,6^. Nonetheless, these biologically active materials, apart from Ti, face limitations regarding market approval and clinical application.

Several studies have demonstrated that altering the structural morphology of Ti alloy surfaces can improve their bioactivity, particularly by enhancing bone regeneration and integration^7^. Over time, different treatment methods have been employed to improve bone integration performance on Ti surfaces. These methods include polishing^8^, grit-blasting^9,10^, fine Ti spraying^11,12^, coarse Ti spraying^11,12^, electron-beam melting (EBM) printing^13–16^, and selective laser melting (SLM) printing^17^. While polishing, grit-blasting, and Ti spraying are considered traditional surface treatment methods, EBM and SLM have emerged alongside additive manufacturing (AM). AM, also known as three-dimensional (3D) printing, is a technique that constructs objects layer by layer based on 3D model data. SLM and EBM are two representative AM techniques^18^. SLM employs a fiber laser as its energy source^19^, operating within a chamber filled with inert gas, aiming to reduce oxygen to the maximum extent to ensure high purity and minimize hydrogen absorption. On the other hand, EBM, another technology in metal AM, uses an electron beam as its energy source and takes place in a vacuum chamber^20^. This provides an oxygen-free environment for the process, guaranteeing high purity and reducing the risk of hydrogen absorption. This is especially beneficial when manufacturing parts made of titanium-6aluminium-4vanadium (Ti6Al4V) as it ensures a lower control of impurity elements. Additionally, maintaining the chamber temperature at about 700 °C during fabrication helps reduce residual stresses, thereby decreasing the risk of part distortion and warping. Due to these differences, implants manufactured by SLM might have unmelted or semi-melted raw material powders, and SLM-manufactured implants might also possess residual stresses. Compared to traditional manufacturing methods, 3D printing provides more regular surface structures and superior pore connectivity, which are crucial for effective bone integration^21^.

Because of its AM method using metal powder, 3D printing requires higher post-processing standards than traditional processes^22^. Both EBM- and SLM-printed products contain metal powder residues, which, if not properly processed on time, can cause severe damage to human bone tissue. No comprehensive studies have investigated the impact of surface Ti alloy on preparation and post-processing technologies on the effectiveness of bone repair.

Currently, our research team mainly employs a process involving high-pressure water jet cleaning, dry ice spraying, and additional cleaning to manage these powders. However, it is necessary to further investigate the impact of this treatment method on the surface structure, mechanical properties, and wear resistance of implants.

This study prepared Ti alloy implants for each group using polishing, grit-blasting, Ti spraying, EBM printing, and SLM printing. The influence of the manufacturing process, post-processing technology, and other processes on parameters such as osteogenic activity, bone integration efficiency, and mechanical strength of the implant was investigated. This research is significant for guiding the preparation, post-processing, and clinical application of Ti alloy implants.

## Materials and experimental methods

### Ethical approval

Each author certifies that his or her institution has approved the animal protocol for this investigation and that all investigations were conducted in conformity with ethical principles of research. Animal experiments were approved by the Ethics Committee of the People's Hospital of Peking University and all experiments were performed in accordance with relevant guidelines and regulations. All animal experimental procedures were performed in accordance with ARRIVE guidelines^23^. This study was performed in accordance with the Declaration of Helsinki. Informed consent for the study was obtained from all donors.

### Material preparation

Researchers designed computer models of EBM and SLM implants (10 mm in diameter and 2 mm in height) in-house. The 3D-printed lattice unit was designed with a rhombic hexagonal structure, and the implant was formed by regular stacking of this lattice unit. Commissioned Suzhou Kangli Orthopaedic Instrument Co., Ltd (China) to perform the printing and post-processing using grade 5 Ti alloy^24^. The EBM printer was an Arcam A1 (Arcam, Sweden), and the SLM printer was an FS121M-D (Hunan Huasheng Gaoke, China). Implant-grade Ti6Al4V (10 mm in diameter and 2 mm in height) was purchased from Beijing Lidakang Technology Co., Ltd. (China) and surface-treated (polished, grit-blasted, fine Ti-sprayed, and coarse Ti-sprayed). These four groups were selected as representatives of traditional processes and served as a basis for comparing the products generated through AM. To explore the effect of the printing powder on the human bone marrow mesenchymal stem cells (hBMSCs) biology, the SLM-printed group was further categorized into three groups: post-treatment (PT-SLM), non-post-treatment (NPT-SLM), and no powder (NP-SLM). The non-post-treatment group underwent ultrasonic cleaning once after printing. In the post-treatment group, ultrasonic cleaning was followed by excess powder removal using dry ice blasting. The laser power was increased to produce no-powder SLM, and the printing speed decreased during manufacturing. After several dry ice-blasting post-processing treatments, a set of SLM-printed implants without a semi-melted powder was prepared. Compared to traditional grit-blasting, dry-ice blasting evaporates automatically, reducing the likelihood of powder residue. Subsequently, all implants were cleaned thrice using an ultrasonic cleaner. Five samples were prepared for each type of implant.

### Material characterization

The macrostructure of the implant surfaces was observed using a high-definition charge-coupled device (CCD) microscope (GP-660V, Gaopin, China), while the micromorphology was examined using a scanning electron microscope (SEM) (JSM-7900F, JEOL, Japan). The implant was subjected to an accelerating voltage of 10 kV, and the observation magnifications of 30× and 150× were used.

### Cell culture

The hBMSCs culture was performed strictly according to the policies of the Ethics Committee of Peking University People's Hospital. Prior informed consent was obtained from the bone marrow blood donor (31 years old, female). The cells were then treated using stem cell enrichment technology. To initiate culture, 5 mL of bone marrow blood was mixed with 10 mL of Minimum Essential Medium (MEM, Hyclone, Tauranga, New Zealand) containing 10% fetal bovine serum (FBS; Gibco Laboratories, Gaithersburg, MD, USA) and 1% double antibodies (Hyclone, Tauranga, New Zealand) in a 10 cm cell culture dish. The cells were cultured in a constant-temperature incubator at 37 °C and 5% CO_2_. Every 5.5 days, half of the culture medium was replaced. After the third replacement, the full culture medium was performed every 2 days. Cells were observed under the microscope every day, and by day 21, the cell confluence at the bottom of the culture dish reached 80%. The cell culture dish was washed with PBS, and the cell adhesion was observed under the microscope. The cells adhered to the bottom of the culture dish were P0 generation hBMSCs. After subculturing to P3, they will be used for subsequent experiments.

### Cell adhesion, proliferation, and osteogenic differentiation

#### Adhesion

The hBMSCs (1 × 10^4^ cells/mL) were cultured with each implant group. After 4 h of incubation, non-adherent cells were washed away with PBS (Gibco Laboratories, Gaithersburg, MD, USA). Adherent cells were fixed in 2.5% glutaraldehyde for 20 min. Following a series of gradient ethanol dehydration steps, the cells were observed and counted using an SEM.

#### Proliferation

Cell Counting Kit-8 (CCK-8, Dojindo, Japan) was used to assess the proliferation of hBMSCs on different surfaces. Cells (1 × 10^4^ cells/mL) were cultured on each sample for 1, 3, or 7 days. At the end of each period, the culture medium was replaced with 500 μL of fresh medium and 50 μL of CCK-8 solution. After a 2-h incubation, the supernatant was transferred to a new 96-well plate. The absorbance was measured at 450 nm using a microplate reader (Bio-Rad, USA). All experiments were repeated thrice.

#### Osteogenic differentiation

The hBMSCs (1 × 10^4^ cells/mL) were cultured with each group of implants in 24-well plates; culture wells without implants were set as the blank control group. The medium was changed to osteogenic-induced differentiation medium (HUXMA-90021, Cyagen, China) after cell adhesion (the next day). After 7 days of culture, the medium was aspirated, the implants were washed with PBS three times, and the implants were transferred to a new 24-well plate. Proteins were extracted according to the instructions of the ALP semi-quantitative kit (P0321, Beyotime, China), and the absorbance at 450 nm was detected by an enzyme marker after success.

After using the same culture method as the ALP semi-quantitative assay for up to 28 days, the implants were fixed with 4% paraformaldehyde for 30 min at room temperature, washed three times with PBS, and then alizarin red staining solution (Cyagen, China) was added. The implants were incubated at room temperature for 30 min, after which the implants were washed repeatedly with PBS until the PBS was clarified. The formation of calcium nodules on the implant surface was then photographed using a high-definition CCD microscope. Thereafter, 10% cetylchloropyridine solution (C9002-25G, Sigma, USA) was used to dissolve the calcium nodules. The absorbance at 562 nm was detected using an enzyme marker, and the data were collected and statistically analyzed.

### Mechanical analysis of 3D-printed implants

In accordance with the experimental program of GB/T 7314-2017, an Instron-5569 testing machine (Instron, Massachusetts, USA) was used to test the compressive strength of EBM, PT-SLM, NPT-SLM, and NP-SLM, and each group of tests was repeated three times. Data were collected and statistically analyzed.

### Establishment of the animal model

Sixty-three adult female New Zealand White rabbits weighing between 2.5 and 3.0 kg were divided into seven groups: polishing, grit-blasting, fine Ti spray, coarse Ti spray, EBM pressure, PT-SLM pressure, and NPT-SLM pressure. The groups were divided into 3 groups of 3 rabbits each, depending on the sampling time. All animal experiments were conducted in accordance with the Guidelines for Ethical Review of Animal Welfare for Laboratory Animals (GB/T 35892-2018) and approved by the Ethics Committee of the People's Hospital of Peking University. After routine anesthesia for skin preparation, lumianning was injected intravenously at the ear margin, and cefazolin 250 mg was injected intramuscularly to prevent infection. The skin of the right lower limb was sterilized, and an incision of approximately 1 cm was made lateral to the distal end of the right femur, bluntly dissecting the soft tissues to expose the lateral bony surface of the femoral condyle. A cylindrical hole of 5 mm diameter and 10 mm depth was drilled with an orthopedic drill, and the stent was implanted. When the top of the scaffold was aligned with the surrounding bone surface, the wound was irrigated with saline, and the incision closed. After surgery, the animals were observed daily for complications and were allowed to move freely and eat normally. At 4, 8, and 12 weeks postoperatively, the rabbits were subjected to general anesthesia by intramuscular injection of xylazine/ketamine, and the corresponding groups were killed by intracardiac injection of an overdose of sodium pentobarbital. The right femur was removed intact according to the original incision approach, and after the removal of soft tissue on the bone surface, the distal femur was removed and fixed in 4% paraformaldehyde. Alizarin red was injected 2 weeks before, and calcium xanthophyll fluorescence dye was injected 1 week before each harvest.

### Radiological analysis

Micro-computed tomography scanning and reconstruction were performed on three specimens from each group. Bone trabecular thickness (Tb.Th), bone trabecular number (Tb.N), bone trabecular relative volume (BV/TV), and bone trabecular space (Tb.Sp) were measured and recorded. Results were recorded and counted.

### Histological analysis

After fixation for 7 days, the specimens were rinsed in water, dehydrated through an ethanol gradient (70% to 100%), and embedded in a single posterior monomer (methyl methacrylate, MMA). Sections were stained with Masson's trichrome and Van Gieson **(**VG). Sections were collected using a microscope (Leica, Germany), and images were processed using Image J to derive the percentage of bone-implant contact (BIC%); data were collected and analyzed statistically.

### Statistical analysis

All experimental data are presented as the mean ± standard deviation. Differences among groups were evaluated using one-way analysis of variance (ANOVA) followed by Tukey's post hoc test. The statistical analysis was performed using SPSS software (version 25.0; IBM Corp. USA). Statistical significance was set at P < 0.05.

## Results

### Material characterization

**Figure 1:**
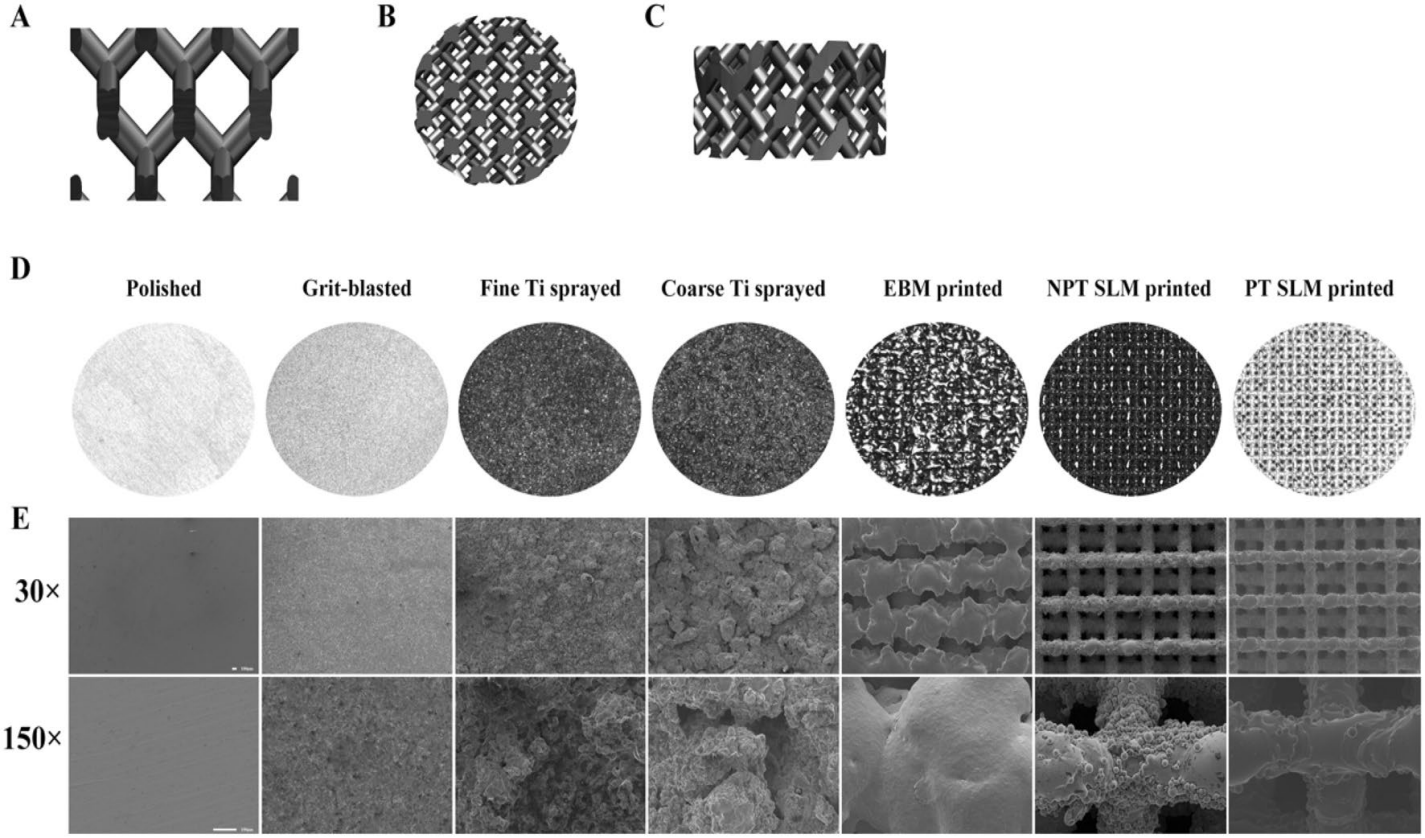
(**A**) A 3D-printed porous structure unit. (**B**) The printed top view of the implant and (**C**) the side view. (**D**) Stereo microscope photographs of the general view of the surface of each implant. (**E**) The surface microstructure of the implants was observed by SEM.

The computer design structure of each group of 3D-printed samples was diamond-shaped (Fig. 1A–C). The polished group exhibited a smooth surface with very tight and unnoticeable polishing. The blasting treatment method revealed residual structures resulting from the impact of tiny particles observed under 30× magnification and distributed more evenly under 100×. The fine Ti particles ranged from 100 to 300 mesh and appeared as small, disordered particles on the material surface, evenly distributed under high magnification. The coarse Ti particles ranged between 40 and 80 mesh and showed larger disordered particle structures, approximately twice the size of the fine Ti spray. Under high magnification, a stronger 3D sense of the coating structure was observed, characterized by many uneven "grooves." However, both EBM and SLM in the 3D-printed group formed multilayer uniformly ordered porous structures with a 400–500 um pore size. Under 30×, the structure formed by SLM exhibited greater regularity than that formed by EBM. However, at 100×, the surface of the EBM group was smoother than that of the SLM group, indicating that the scaffold of the EBM group was easier and more thoroughly cleaned after printing. The PT-SLM and NPT-SLM surfaces contained residual material particles and semi-melted materials, resulting in rough surfaces. In the PT-SLM group, the particles were mostly semi-melted and adhered to the scaffold, becoming part of it. The NPT-SLM group contained more free powder (Fig. 1D and E).

### Cell adhesion and proliferation

**Figure 2:**
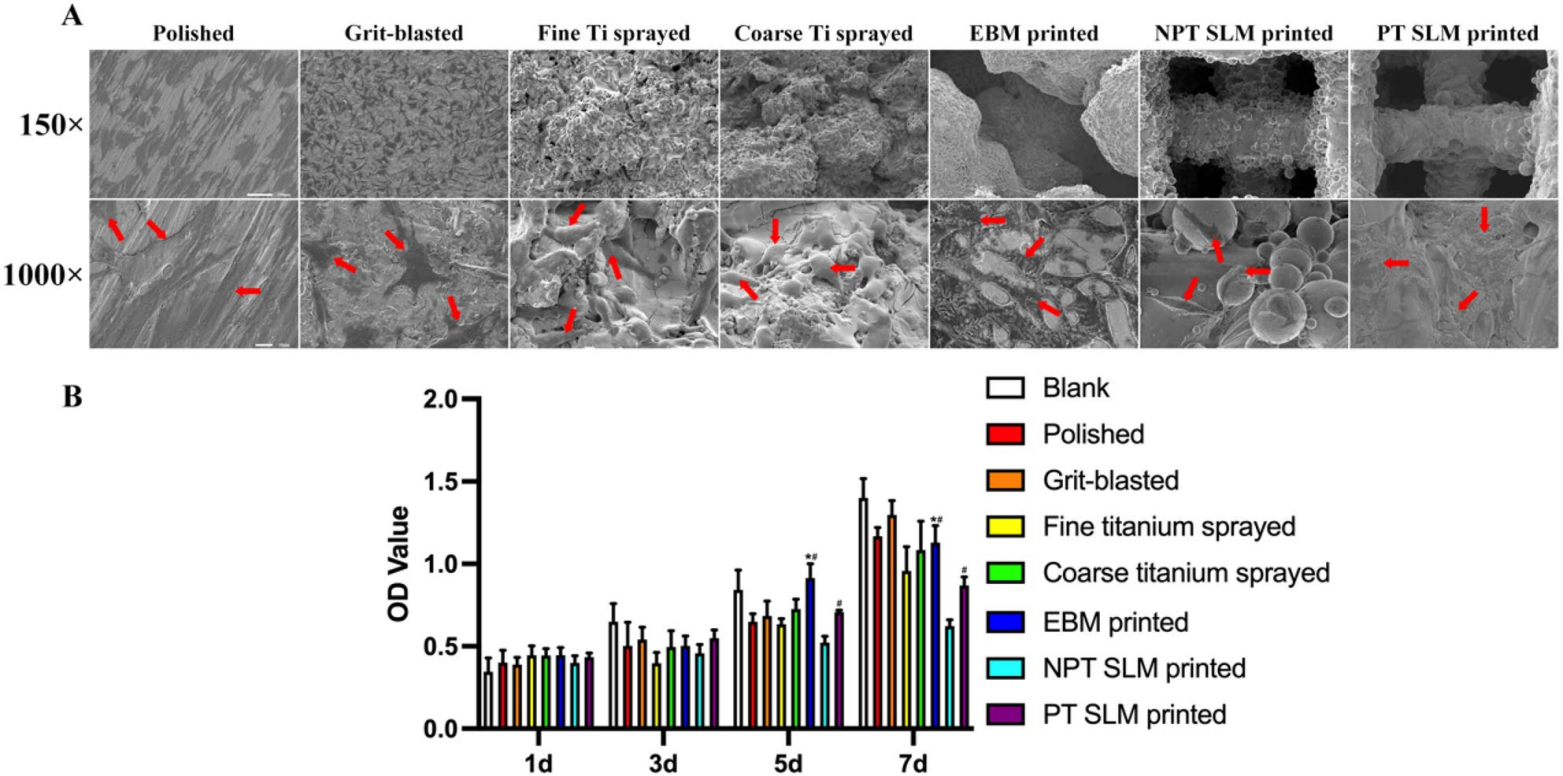
(**A**) Number and morphology of hBMSCs adhering to the surface of implants after 7 days of culture. The red arrow indicates hBMSCs. (**B**) The activity of hBMSCs on the surface of implants in the 7 groups. *P < 0.01 EBM compared with the PT-SLM. ^#^P < 0.01 EBM compared with the PT-SLM.

The hBMSCs were cultured with seven different implants, and the CCK-8 values of the samples were measured at 1, 3, 5, and 7 days to assess cell proliferation. After 7 days, the implants were fixed, and cell morphology was observed under an electron microscope. In the traditional treatment group, the blasting group exhibited the highest absorbance, with an optical density (OD) value of approximately 1.3 on the 7th day. In the 3D-printed group, EBM was significantly stronger than SLM, with an OD value of approximately 0.8 for PT-SLM and 1.1 for EBM on the 7th day (statistically significant compared to PT-SLM, P < 0.05). Furthermore, PT-SLM demonstrated significant improvement over NPT-SLM (Fig. 2B). Under an electron microscope, it was found that in the traditional treatment group, the cells in the fine Ti spray group were in a better state. These cells exhibited spreading, increased volume, polygonal, and obvious pseudopodia. In contrast, the polishing group showed the poorest cell state, with minimal cell coverage on the surface and no pseudopodia. In the 3D-printing group, the NPT-SLM group had the worst cell state, characterized by sparse cells, poor shape, and absence of obvious spreading or pseudopodia. However, the EBM group exhibited greater cell coverage on the surface and a higher presence of more pseudopodia (Fig. 2A).

### Osteogenic differentiation

**Figure 3:**
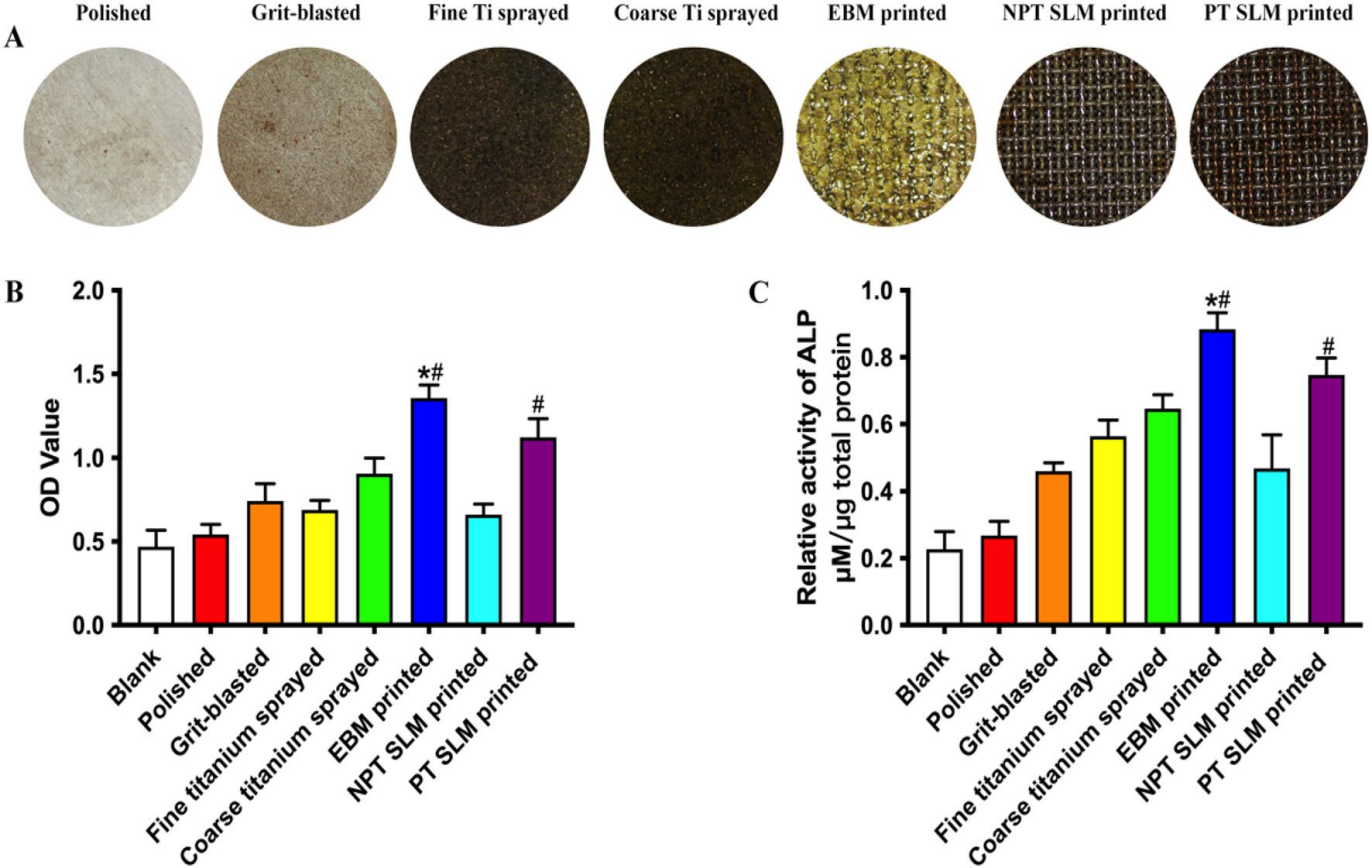
(**A**) Formation of calcium nodules on the surface of plants stained with alizarin red. (**B**) Semi-quantitative analysis of dissolved calcium nodules. (**C**) Semi-quantitative analysis of ALP. *P < 0.01 compared with the SLM-printed (post-processing) group. ^#^P < 0.01 compared with the SLM-printed group.

The implants cultured with hBMSCs were stained with alizarin red, resulting in the observation of red–black calcium nodules on the surface of the implants. Within the traditional treatment group, the surface of the fine Ti spray exhibited a significantly higher presence of calcium nodules than the other three groups. In the 3D-printed group, the NPT-SLM surface displayed the lowest number of calcium nodules, whereas the EBM group had the highest. Semi-quantitative analysis was performed to quantify the number of calcium nodules. In the 3D-printed group, the absorbance of EBM and PT-SLM was significantly higher than that of the conventional treatment group, with an OD value of 1.4 EBM and 1.1 PT, which was statistically significant (P < 0.05). These findings indicate that the 3D Ti material printed using EBM promotes the calcification of hBMSCs more effectively (Fig. 3A,B). The semi-quantitative results of ALP showed that the EBM group had a value of 1.1 uM/ug, significantly higher than the remaining six groups. Specifically, it showed statistically significant compared to PT-SLM (P < 0.05), indicating its effective promotion of early osteogenesis. The NPT-SLM group exhibited poor results in both experiments, indicating the presence of residual-free powder in the material significantly hindered cell proliferation and differentiation (Fig. 3C).

### Mechanical analysis of 3D-printed scaffolds and cell adhesion of over-cleared powder scaffolds

**Figure 4:**
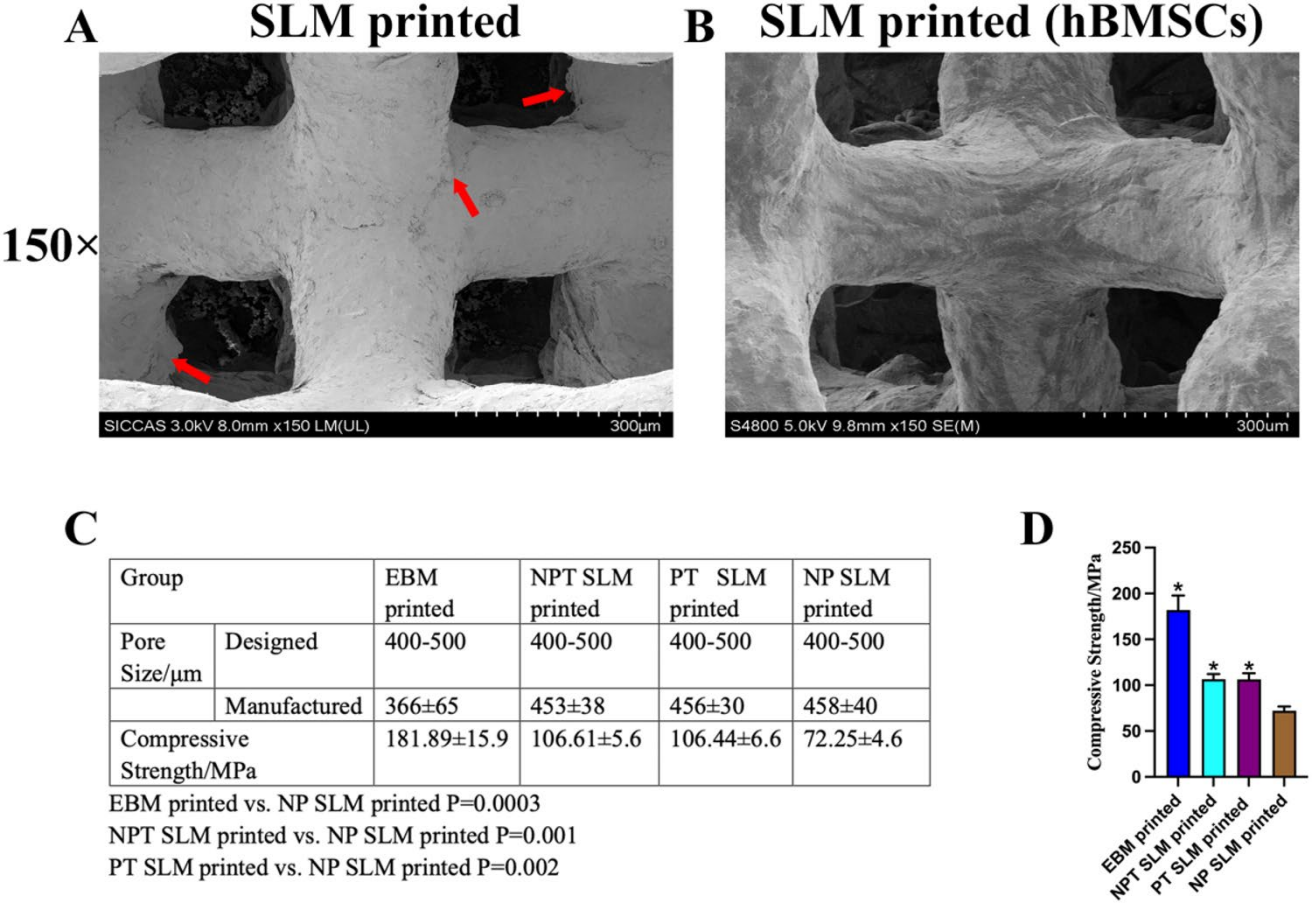
(**A**) A porous implant with no powder residue on the surface prepared by varying the SLM printing parameters. (**B**) The morphology of hBMSCs adhering to the surface of implants after 7 days of cultivation. (**C**) Compressive strength test of EBM and SLM-printed groups. (**D**) Statistical graph of compressive strength of each group. EBM-printed vs. SLM-printed (no powder) *P = 0.0003; SLM-printed vs. SLM-printed (no powder) *P = 0.001; SLM-printed (post-processing) vs. SLM-printed (no powder) *P = 0.002.

The 3D-printed scaffolds, produced through the commonly used SLM manufacturing process, inevitably produce semi-melted particles, and it is difficult to remove the residual powder particles completely. However, scaffolds without powder were obtained by increasing the printer power and reducing the laser speed (Fig. 4A). Under a 150× electron microscope, it is evident that compared with the commonly used SLM, the cells on the surface of the NP-SLM spread better and had more pseudopodia (Fig. 4B). However, the mechanical properties of the scaffolds obtained by this method were inferior to those of EBM, NPT-SLM, NP-SLM, and PT-SLM, indicating that this biological advantage was achieved at the expense of the mechanical properties (Fig. 4C). Statistical analysis of the compressive strength of each group revealed that EBM-printed vs. SLM-printed (no powder), SLM-printed vs. SLM-printed (no powder) and SLM-printed (post-processing) vs. SLM-printed (no powder) were all statistically significant (Fig. 4D).

### Radiographic analysis

**Figure 5:**
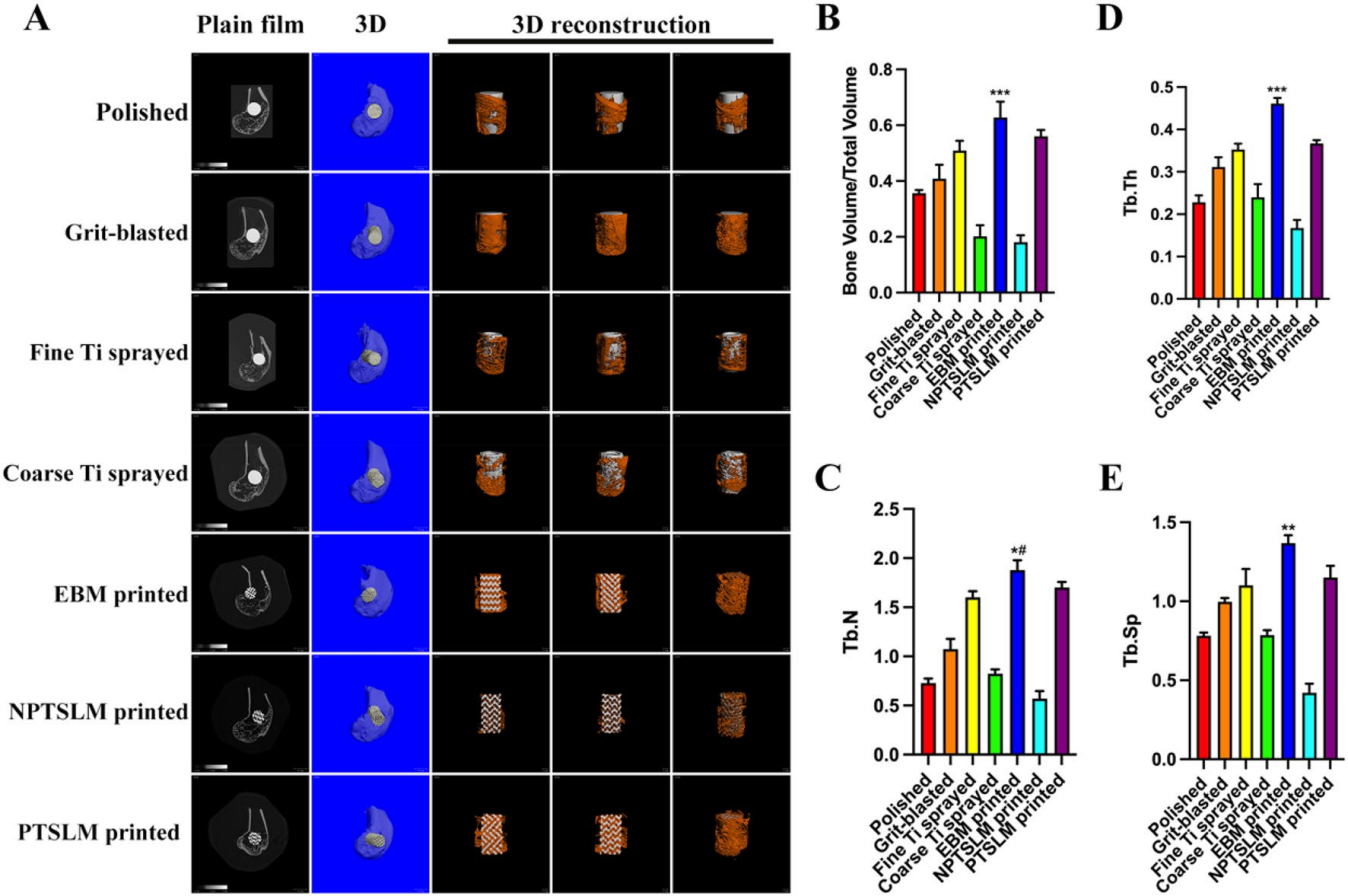
(**A**) Micro-CT assessing osteogenesis of implants in the rabbit femoral condyle using different processing methods; (**B**) Semi-quantitative analysis of bone mass through Bone Volume Fraction (BV/TV). EBM-printed vs. Fine Ti-sprayed ***P < 0.001; (**C**) Trabecular number (Tb.N) assessing the spatial morphology of trabecular structure. EBM-printed vs. Fine Ti-sprayed *#P < 0.01; (**D**) Trabecular thickness (Tb.Th) assessing the spatial morphology of trabecular structure. EBM-printed vs. Fine Ti-sprayed ***P < 0.001; (**E**) Trabecular separation (Tb.Sp) assessing the spatial morphology of trabecular structure. EBM-printed vs. Fine Ti-sprayed **P < 0.01.

After the scaffolds in each group were implanted into New Zealand rabbits for 12 weeks, they were harvested for micro-CT analysis. The results demonstrated that among the traditional manufacturing methods, the materials used for blasting and fine Ti spraying had better bone regeneration and integration effects. In contrast, the bone regeneration effects of the polishing and coarse Ti-sprayed groups were not ideal. In the AM group, the scaffold printed with EBM exhibited the best bone regeneration, integration, and growth performance (Fig. 5A). The semi-quantitative analysis results showed that the EBM group had the highest BV/TV value compared to all other groups, with a significant difference observed when compared with the fine Ti group using the traditional manufacturing method (Fig. 5B, ***P < 0.001). In addition, regarding bone regeneration quality, the Tb.N, Tb.Th, and Tb.Sp values in the EBM group were the highest among all the groups (Fig. 5C–E).

### Histological analysis

**Figure 6:**
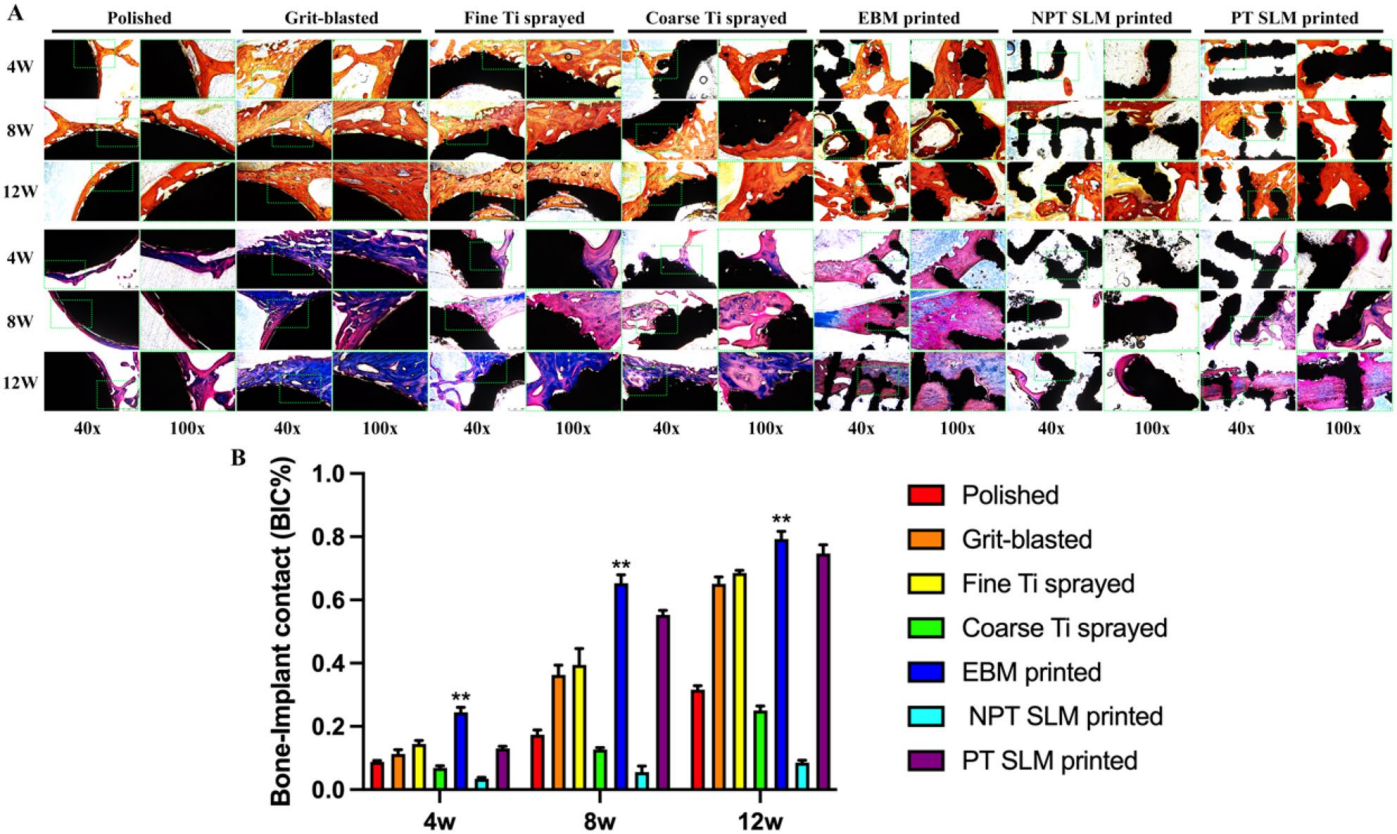
(**A**) Hard tissue sections were taken from the rabbit femoral condyle implanted with different treatment implants at 4 weeks, 8 weeks, and 12 weeks, then VG and Masson staining was performed to assess the impact of different implants on bone regeneration and bone integration, and the results showed that the EBM method had the best effect; (**B**) Bone implant contact percentage (BIC%) was used to evaluate the ability of different implants for bone regeneration and bone integration. The data showed that EBM had the highest scores at 4 weeks, 8 weeks, and 12 weeks, and the difference was statistically significant, **P < 0.01.

**Figure 7:**
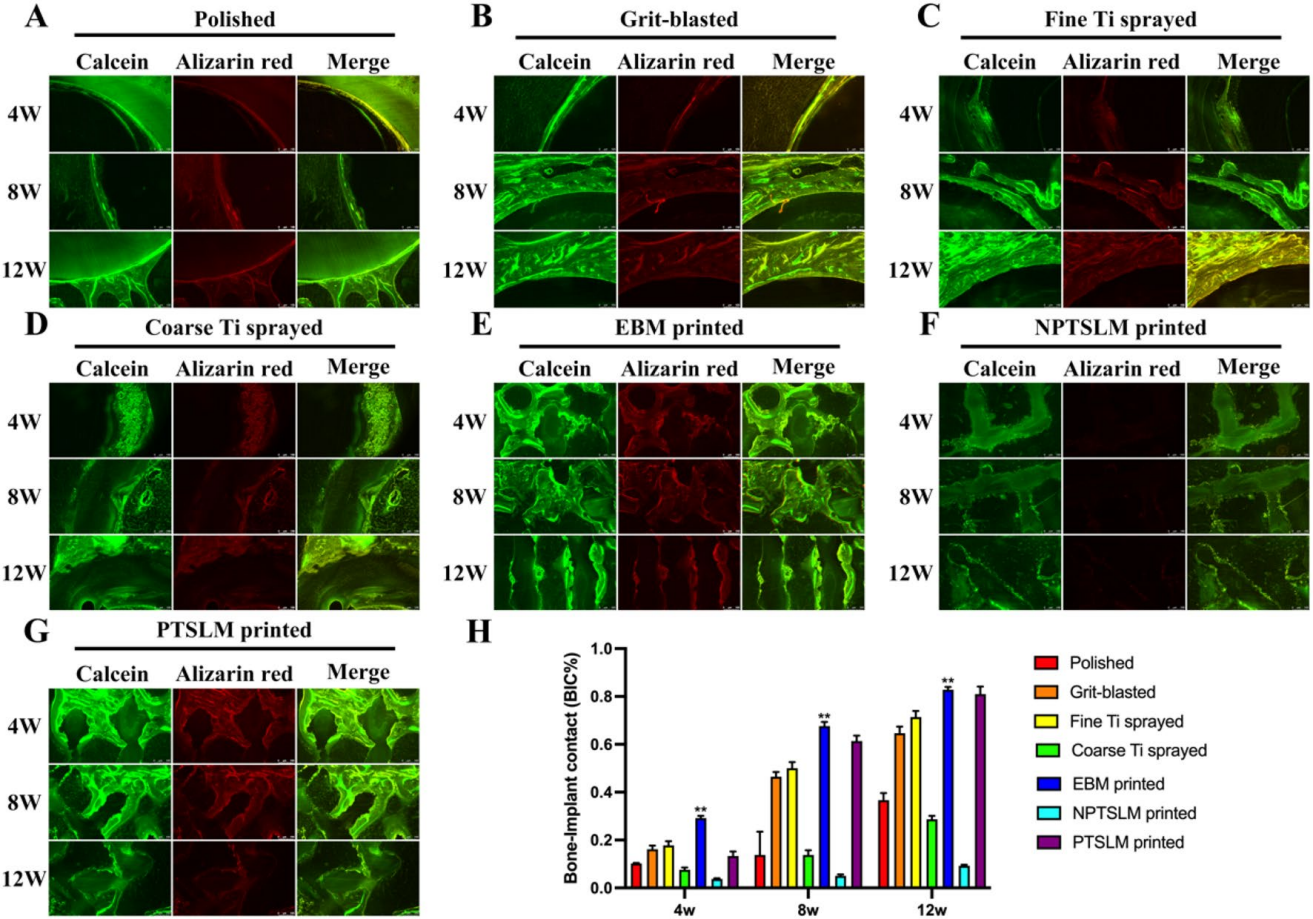
(**A**–**G**) Hard tissue sections were stained with calcein-alizarin red to assess the effect of different implants on bone regeneration and bone integration; (**H**) Bone implant contact percentage (BIC%) was used to evaluate the ability of different implants for bone regeneration and bone integration. The data showed that EBM had the highest scores at 4 weeks, 8 weeks, and 12 weeks, and the difference was statistically significant, **P < 0.01.

Samples were collected for hard tissue sectioning at 4, 8, and 12 weeks, followed by VG and Masson staining. In the traditional treatment group, fine Ti spray bone regeneration and integration effects were optimum, whereas bone regeneration, integration, and growth of the EBM were optimum in the 3D-printed group (Fig. 6A). The BIC% shows that the BIC value of fine Ti spraying in the traditional treatment method is the highest, indicating that its induced integration ability is stronger than the other three groups. At 4, 8, and 12 weeks, the EBM values were 0.22, 0.65, and 0.80, respectively, higher than the other six groups and statistically significant (P < 0.01). The NPT-SLM demonstrated the least effective results (Fig. 6B). Fluorescent double-labeling results showed that the amount of regenerated bone and integration efficiency in the polished group were low (Fig. 7A). The grit-blasted group exhibited good bone regeneration and integration performance (Fig. 7B). However, its performance group in vivo was not as effective as that of the fine Ti spray group (Fig. 7C). In the coarse Ti spray and NPT-SLM groups, many free Ti particles were observed in the image indicating poor bone integration performance (Fig. 7D,F). There was a significant amount of bone tissue inside the EBM and PT-SLM scaffolds and solid integration (Fig. 7E,G). The BIC analysis results revealed that the EBM group performance was the best among the seven groups, with a value significantly different from that of the fine Ti spray group (Fig. 7H, **P < 0.01). However, at 12 weeks, no significant differences were observed between the EBM and PT-SLM groups.

## Discussion

The reconstruction of bone defects that remain after the excision of skeletal lesions has always been the focal point in orthopedics. Metal prosthesis reconstruction has remained the predominant solution^25^. Therefore, the efficiency of integration between metal and bone interfaces is crucial in determining the likelihood of prosthesis loosening^26,27^. A large body of literature indicates that differences in the metal material surface structure can lead to variations in bone integration efficiency^27–29^. Notably, surface structure influences bone integration behavior and impacts the biological behavior of osteoblasts, including proliferation and osteogenic differentiation, as evidenced by our preliminary research^21^. Osteogenic differentiation and bone integration jointly determine the fate of the implant^30^.

Medical Ti alloys are widely used in clinical practice for implant manufacture because of their excellent biocompatibility, mechanical properties, and corrosion resistance^31,32^. However, Ti alloys lack inherent biological activities. Ti alloys readily form an oxide film when inside the human body. If implants do not undergo surface treatments, they can easily become loose^33,34^. Hence, to prolong the implant lifespan, researchers and device manufacturers have attempted to treat the surface of the implants to create rough structures that promote firm bonding between bone and metal.

Several surface-treatment methods are available for Ti alloys. Currently, the most common methods include polishing, grit-blasting, Ti spraying, and 3D spatial structures obtained using 3D printing technology (EBM and SLM printing). These manufacturing methods have already been used in preparing clinical prostheses. However, each method possesses its strengths and weaknesses. The polished group displayed a smooth surface, which hinders osteoblast adhesion. This is evident from the in vitro cell experiment results, which showed fewer adherent cells in the polished group and an unsatisfactory spreading effect. Conversely, the grit-blasted group exhibited better quantity and condition of cell adhesion.

Grit-blasting involves creating rough depressions on the implant surface through "impact" in addition to the polishing process. The significance of the spatial form may not be particularly pronounced. However, even these subtle changes in the spatial structure can alter the biological behavior of cells, further corroborating the importance of spatial structures. Among all the experimental groups, the coarse Ti spray-coated group stands out. However, there were discrepancies between the in vitro and in vivo experimental results of this group. In vitro, the coarse Ti spray provided the most 3D spatial structure, apart from the 3D-printed group. The experimental results confirmed comparable cell proliferation and adhesion rates with the 3D-printed spatial structure. However, the radiographic and histological experimental results in vivo were disappointing.

In particular, the histological section observation revealed that coarse Ti particles were scattered in the bone tissue, significantly affecting bone regeneration and integration. This phenomenon could be attributed to in vivo corrosion and wear. Comparing the experimental results, these scattered Ti particles, similar to the uncleaned free powder of the NPT-SLM group, severely impeded bone regeneration and integration. This finding reminds clinicians and prosthesis manufacturers about the importance of conducting a coating adhesion test before using a coated prosthesis to ensure that excessive shedding does not occur during in vivo service, which could lead to severe consequences.

In the first phase of this study, in vitro experiments were conducted, including the cultivation of differently treated materials with hMSCs and osteogenic induction tests. In the cultivation experiment, cell morphology and viable cells were determined using electron microscopy and CCK-8 assays. The electron microscopy scanning results revealed that, in comparison to traditional treatment methods, the cell conditions of the 3D-printed group were better, covering more of the implant surface, with more cells spreading and a greater number of pseudopodia, with EBM being superior to PT-SLM. The results of the fine Ti spray group were better than those of the other groups (polishing, grit-blasting, and coarse Ti spraying). In the CCK-8 test, the OD value of the grit-blasted group was the highest, possibly because of the experimental error. This discrepancy could arise because Ti-sprayed and 3D-printed implants contain numerous voids. Before the addition of the CCK-8 reagent, some culture medium may reside in these voids, resulting in a smaller absorbance because some cells do not come into contact with the solution.

Alizarin red staining, semi-quantitative calcium nodule test, and ALP staining were conducted in the osteogenesis test. The results demonstrated that the 3D-printed group outperformed the traditional treatment group and showed statistically significant differences, with EBM being superior to PT-SLM, indicating that EBM printing is currently the most suitable printing technology for clinical use.

The second phase involved in vivo experiments analyzing the bone regeneration and integration performance of each group through radiology and histology over a maximum in vivo service period of 12 weeks. The overall results were consistent with the findings from the in vitro experiments. The EBM and PT-SLM groups exhibited superior performance to the traditional treatment groups. Among the traditional treatment groups, fine Ti spraying was superior to the other treatments. Within the 3D-printed groups, EBM was superior to PT-SLM.

In this study, we observed an interesting phenomenon. In the in vitro cell proliferation experiment, both the grit-blasted and polished groups performed well. However, in the osteogenic differentiation experiment, these two groups did not perform as well as the others. The results of the osteogenic differentiation experiments are consistent with those of the in vivo experiments. This suggests that the spatial structure indeed affects the process of stem cell differentiation towards bone and that merely promoting cell proliferation cannot determine the final outcome of bone regeneration. An appropriate spatial structure is the final determinant of the efficiency of bone regeneration and integration of an implant in vivo, thereby influencing the implant's lifespan. However, what is a suitable spatial structure for measurement? Previous researchers have emphasized the importance of surface morphology and roughness of the implant for bone integration^35^, as rough surfaces promote the adhesion and proliferation of osteoblasts^20,31,32,36–44^. However, there is currently no consensus on the optimal roughness.

This study demonstrated that the in vivo and in vitro experiment results of the 3D-printed group with regular spatial structure were better than those of the traditional coating preparation group. These findings suggest that regular spatial structures, interconnected pores, and a certain pore depth can achieve better osteogenic differentiation behavior. Due to the excellent osseointegration ability of Ti alloys, they have become one of the materials used for orthopedic implants. With the advent of 3D printing technology, there are now multiple shape options for implants. 3D-printed Ti alloy implants are widely used in bone defects caused by trauma, infection, and tumors^45–48^.

Additionally, considering the mechanical properties, Ti alloys have an elastic modulus of approximately 114 GPa, while Mechanical stiffness ranges from 14.7 GPa to 34.3 GPa for cortical bone^49,50^ and 0.001 GPa to 2.942 GPa for cancellous bone^50^. In healthy individuals, bones undergo remodeling based on the mechanical pressure they endure. However, owing to the high modulus of Ti, the stress transferred to the adjacent bone is reduced, leading to bone absorption and aseptic loosening of the implant. This effect, caused by a relative hardness difference called "stress shielding," significantly affects the implant's lifespan. Implants prepared by 3D-printing technology alleviate the stress-shielding effect to some extent. Currently, mainstream technologies, such as EBM and SLM, enable precise control over the pore size and structure of porous Ti alloys^51,52^. This control results in alloys with a relatively large surface area and complex pore structure. Interconnected pores are more conducive to bone and vascular ingrowth and nutrient exchange than isolated pores. Consequently, these alloys have mechanical properties similar to those of human bones (Supplementary Table 1), which significantly enhances the effect of bone integration, transforming Ti alloys into high-performance, bioactive, and mechanically stable prostheses^20,37^.

EBM and SLM are AM (3D printing) techniques for producing metal parts, particularly for Ti and its alloys. Although both are based on the principle of layer-by-layer melting of a powder bed to construct the final product, several key differences exist between the two methods, with the primary difference being the heat source. SLM employs a high-power laser beam^19^ as its energy source, whereas EBM utilizes a high-energy electron beam^53,54^ with even greater power. Another difference is the manufacturing environment; while SLM typically operates in an inert gas atmosphere, usually argon or nitrogen, to prevent material oxidation, EBM operates in a vacuum chamber^20^. This means that there is an absence of oxygen and other impurities, offering better protection to the metal components compared to the inert gas environment of SLM. This leads to differences in the implants produced by the two manufacturing methods. Due to insufficient temperature in SLM, there may be residual raw material powder^55,56^ in the implant, and the rapid and localized heating and cooling during the manufacturing process result in residual stress in the implant, necessitating post-processing^17,57,58^. In contrast, with its higher temperature, EBM ensures complete melting of the raw material, leaving no residual particles in the implant. The stable temperature during the construction process reduces the risk of residual stress and deformation.

In this study, electron microscopy revealed residual Ti particles, semi-melted Ti particles, and free Ti particles on the surfaces of SLM-printed implants. There was no free powder in the post-treatment PT-SLM group; however, a large amount of the powder was in a semi-melted state on the scaffold. We hypothesize that this semi-melted powder is safe and can be considered a part of the scaffold, providing mechanical strength. To test this hypothesis, we increased the laser power, decreased the printing speed, and used high-intensity post-processing to obtain a completely no-powder, smooth scaffold. Although we achieved better cell adhesion effects, this was achieved at the expense of a significant improvement in mechanical performance. In contrast, the EBM group had a virtually zero-free powder, making post-processing easier. Experimental results also confirmed that the free powder increased in vitro osteoblast apoptosis, poor osteogenesis, limited in vivo bone regeneration, and increased osteolysis, ultimately affecting bone integration.

Although cells tend to adhere more to rough surfaces than smooth surfaces, this does not mean that the rougher the implant surface, the better. This finding is consistent with previous studies. Vercaigne et al.^38^ conducted in vivo studies on implants with different surface roughness and found that increased roughness did not lead to increased bone integration but led to higher Ti ion release, which negatively impacted osteogenesis. Batzer et al.^59^ found that greater surface roughness increased the production and secretion of hormones (such as PGE2), affecting the function of cells on the implant surface thereby affecting the implant's long-term stability. Jin et al.^60^ highlighted that the presence of free particles in the implant led to increased secretion of IL-6 and TNF-α by osteoblasts, and that these cytokines are considered predictors of implant loosening or failure. Furthermore, the release of Ti ions hinders the normal function of cells; however, a coarse Ti spray surface stimulates cells to produce hormones that are detrimental to cell adhesion and growth. Further experiments are required to explore the optimal roughness and quantify it as a guideline for clinicians and device manufacturers.

## Conclusions

Surface treatment (grit-blasting and Ti spraying) of planar structures can indeed increase the adhesion of hBMSCs; however, it does not necessarily promote the osteogenic differentiation of hBMSCs. 3D-printed implants with regular pore structures enhance the osteogenic differentiation of hBMSCs. The framework structure of 3D printing may affect the osteogenic differentiation activity of hBMSCs. SLM-printed implants may have residual unmelted metal powder or powder in a semi-melted state. The presence of residual metal powder on the NPT-SLM-printed implants can hinder the osteogenic differentiation process. The surface of PT-SLM scaffolds may still retain some powder in a semi-melted state. Still, these residual powders can be considered part of the scaffold and do not significantly affect cell activity and differentiation. For SLM-printed implants, the pursuit of "no powder" should not be excessive, as this would impair the mechanical properties of the implant. Compared to PT-SLM, implants printed by EBM are superior in promoting cell adhesion, proliferation, and osteogenic differentiation in vitro. The in vivo experiment results further confirm that EBM is better than PT-SLM.

### Supplementary Information

Supplementary Information.

## Data Availability

The datasets used and analyzed during the current study are available from the corresponding author upon reasonable request.
