# Supplementary material for: Efficacy of bone defect therapy involving various surface treatments of titanium alloy implants: an in vivo and in vitro study
Source: Sci Rep. 2023 Nov 17;13:20116. doi: 10.1038/s41598-023-47495-w (PMC10656537; doi:10.1038/s41598-023-47495-w)
Supplement: Supplementary file 1 — Supplementary Information. [file 41598_2023_47495_MOESM1_ESM.docx]

Supplementary material.

According to the experimental plan established by the national standard, using the Instron-5569 testing machine, the compressive strength and elastic modulus of EBM and PT-SLM were tested respectively. The elastic modulus of the material is determined by the stress-strain curve. Each experiment was repeated 3 times.

Supplementary table 1.The elastic moduli of cancellous bone, cortical bone, PT-SLM, and EBM.

| Group | Elastic modulus（GPa） |
| --- | --- |
| PT-SLM | 5.26～6.57 |
| EBM | 11.46～17.6 |
| cortical bone[1, 2] | 14.7～34.3 |
| cancellous bone[2] | 0.001～2.942 |

Reference

1. Carter, D.R. and W.C. Hayes, The compressive behavior of bone as a two-phase porous structure. J Bone Joint Surg Am, 1977. 59(7): p. 954-62.

2. Peterson, J. and P.C. Dechow, Material properties of the human cranial vault and zygoma. Anat Rec A Discov Mol Cell Evol Biol, 2003. 274(1): p. 785-97.
